# Photophysical Exploration of Alectinib and Rilpivirine: Insights from Theory and Experiment

**DOI:** 10.3390/molecules28166172

**Published:** 2023-08-21

**Authors:** Chun Zhang, Yuting Yang, Suya Gan, Aimin Ren, Yu-Bo Zhou, Jia Li, Da-Jun Xiang, Wen-Long Wang

**Affiliations:** 1 School of Life Sciences and Health Engineering, Jiangnan University, Wuxi 214122, China; 2 Institute of Theoretical Chemistry, College of Chemistry, Jilin University, Liutiao Road 2#, Changchun 130061, China; 3 National Center for Drug Screening, State key Laboratory of Drug Research, Shanghai Institute of Materia Medica, Chinese Academy of Sciences, Shanghai 201203, China; 4 Zhongshan Institute for Drug Discovery, Shanghai Institute of Materia Medica, Chinese Academy of Sciences, SSIP Healthcare and Medicine Demonstration Zone, Zhongshan Tsuihang New District, Zhongshan 528400, China; 5 Xishan People’s Hospital of Wuxi City, Wuxi 214105, China

**Keywords:** fluorescence imaging, Alectinib, Rilpivirine, quantum chemistry

## Abstract

Due to the excellent characteristics of fluorescence-based imaging, such as non-invasive detection of biomarkers in vitro and in vivo with high sensitivity, good spatio-temporal resolution and fast response times, it has shown significant prospects in various applications. Compounds with both biological activities and fluorescent properties have the potential for integrated diagnosis and treatment application. Alectinib and Rilpivirine are two excellent drugs on sale that represent a clinically approved targeted therapy for ALK-rearranged NSCLC and have exhibited more favorable safety and tolerance profiles in Phase III clinical trials, ECHO and THRIVE, respectively. The optical properties of these two drugs, Alectinib and Rilpivirine, were deeply explored, firstly through the simulation of molecular structures, electrostatic potential, OPA/TPA and emission spectral properties and experiments on UV-vis spectra, fluorescence and cell imaging. It was found that Alectinib exhibited 7.8% of fluorescence quantum yield at the 450 nm excited wavelength, due to a larger electronic transition dipole moment (8.41 Debye), bigger charge transition quantity (0.682 e) and smaller reorganization energy (2821.6 cm^−1^). The stronger UV-vis spectra of Rilpivirine were due to a larger electron–hole overlap index (Sr: 0.733) and were also seen in CDD plots. Furthermore, Alectinib possessed obvious active two-photon absorption properties ($\delta_{max}^{TPA}$* ϕ = 201.75 GM), which have potential TPA imaging applications in bio-systems. Lastly, Alectinib and Rilpivirine displayed green fluorescence in HeLa cells, suggesting the potential ability for biological imaging. Investigation using theoretical and experimental methods is certainly encouraged, given the particular significance of developing integrated diagnosis and treatment.

## 1. Introduction

In recent years, optical imaging modalities have shown promise in various health-related applications, such as disease diagnosis and optical-guided surgery [1,2]. Compared to traditional methods, fluorescence-based imaging offers several advantages, including non-invasive detection of biomarkers in vitro and in vivo with high sensitivity, fast response times and excellent spatio-temporal resolution [3,4]. Fluorescence-based imaging often relies on the use of chemical tools, which are fluorescent molecules. Small fluorescent molecules are an important kind of fluorescent compound and have the potential to achieve the real-time monitoring of organ function and the visualization of organ-related processes at the cellular level [5]. An excellent small fluorescent molecule should possess the following characteristics: high fluorescence quantum yield, adequate water solubility, fluorophore with good photo-stability and acceptable bio-compatibility [1]. In recent years, more and more fluorescent molecules combined with drugs, aptamers, peptide sequences and other easily modified ligands have been used to develop novel integrated diagnosis and treatment [6,7,8,9]. Along with the increase in reports about the potential UV-vis/fluorescence spectral properties of some (chemotherapy) drugs, research on integrated diagnosis and treatment has attracted widespread attention in the international fields of biology, materials and medicine [10,11,12]. The technology of integrated diagnosis and treatment is able to track the development, occurrence and treatment process of a lesion site (such as cancer) in real time, implement effective and precise treatment, improve the cure effect, reduce side effects, detect differentiation and metastasis for the lesion site and take measures to maximize patient survival and timely recovery rates. However, the strategy of combining fluorophores and suitable ligands faces some limitations, for example, complicated synthesis, poor permeability, low bioavailability and more. Therefore, none are currently used in clinical applications. Following the significant demand of quality of human life, a small organic drug molecule combining near-infrared (NIR) fluorescence imaging has significance for promoting clinical applications of integrated diagnosis and treatment [13,14].

Alectinib and Rilpivirine (shown in Table 1) are two excellent drugs on sale with USD 1.292 billion and USD 964 million of retail sales volume in 2020, respectively [15]. Alectinib is a highly selective, second-generation inhibitor of the tyrosine kinase anaplastic lymphoma kinase (ALK), and is a clinically approved targeted therapy for ALK-rearranged non-small lung cancers (NSCLCs) [16,17,18]. Importantly, Alectinib is also effective for treating brain metastasis of ALK-rearranged NSCLCs, suggesting its high brain penetrance. In 2020, it was reported that Alectinib could provide a personalized maximum benefit for patients with high-grade serous ovarian cancer who are positive for EML4-ALK [19]. On the other hand, Rilpivirine is a new-generation NNRTI and is considered as a recommended or alternative key drug in the current ART guidelines. It exhibited more favorable safety and tolerance profiles compared with Efavirenz in Phase III clinical trials, ECHO and THRIVE [20,21]. The most commonly observed mutation in patients with Rilpivirine-containing treatment failure is E138K [22]. I135T/L, escape mutations from HLA-B*51/52-restricted cytotoxic T lymphocytes, may predispose HIV-1 to harbor E138K upon failure of Rilpivirine-containing ART and the mutation patterns of drug resistance may vary due to baseline polymorphic mutations [22]. Developing small organic drug molecules with NIR spectra could bring about new opportunities for improving disease diagnosis and effective therapeutics. But it is extremely difficult, so it has not yet been reported. In this study, we thoroughly studied the molecular properties and explored the photo-physical luminous mechanism for these two drugs on sale (Alectinib and Rilpivirine) in order to offer more theoretical foundations for traceable drugs and promote the development of integrated diagnosis and treatment.

## 2. Results and Discussion

### 2.1. Molecular Structural Characteristics

**Figure 1:**
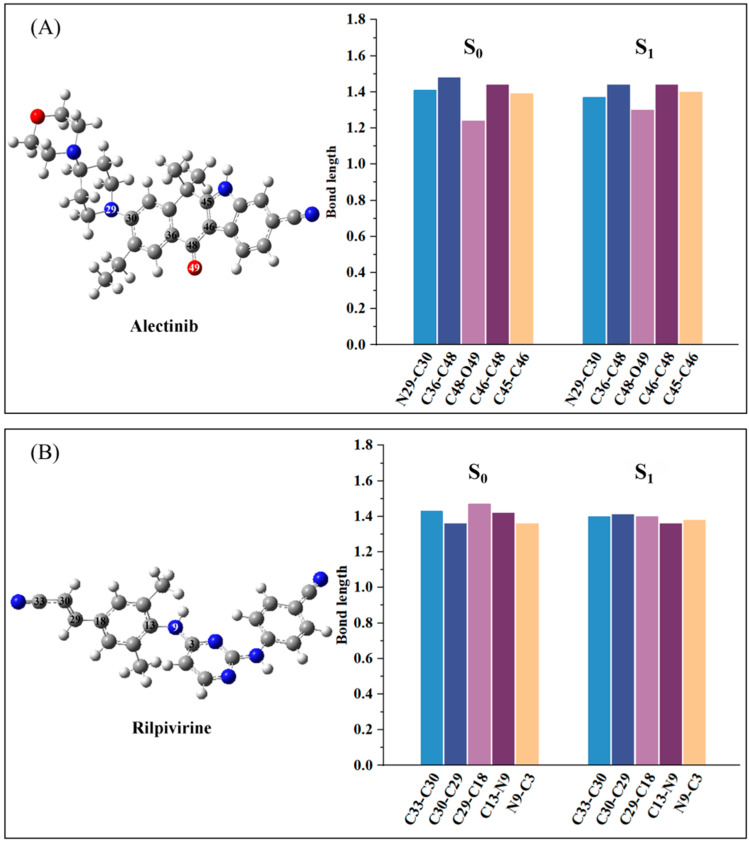
The important bond length (Å) of molecules Alectinib (**A**) and Rilpivirine (**B**) in S_0_ and S_1_.

Firstly, the geometric characteristic is very important to study the electronic and photo-physical properties of a compound. In order to learn the geometric characteristics, the structural parameters of stable ground state (S_0_) and first excited state (S_1_) including the main bond lengths (Å) and dihedral angles (°) were calculated for drugs Alectinib and Rilpivirine, and the resulting data were listed in Table 1 and Table S1. From Table 1 about the geometries from molecular top and side view, it can be seen that in not only S_0_ but also S_1_, molecule Alectinib had a π-conjugation planar molecular skeleton besides the terminal 4-(piperidin-4-yl) morpholine group. And the dihedral angle (see Table S1) between parent molecular plane and 4-(piperidin-4-yl) morpholine group for Alectinib was decreased from the ground state (at about 61.2°) to the excited state (at about 46.6°), suggesting the larger planarity in the excited state. To make the bond length change more intuitive from S_0_ to S_1_, the data about bond length in Table S1 were drawn in Figure 1. As expected, the single bond in S_1_ was shorter (such as C36-C48: 1.48 > 1.43 Å) and the double bond was longer (such as C48-O49: 1.24 < 1.30 Å) than those in S_0_, implying the bond length alternation was smaller in S_1_ (seen Figure 1A), and further implying the enhanced π-conjugation effect in S_1_. In addition, Alectinib possessed strong electron-withdrawing group (–CN) connected with π-conjugation indole group directly. It may lead to marked intramolecular charge transfer (ICT) and benefit for electron transition. The drug molecule Rilpivirine had two conjugated planes, which were distorted by about 145.8° in S_0_ and decreased to about 111.0° in S_1_ (see Table S1), likely due to the steric effect from two methyl substituents on the benzene ring. The two terminal electron acceptors (–CN) in Rilpivirine also were connected with a π-conjugation benzene ring, which maybe benefit electron transition. The same for the molecule Rilpivirine, the important bond length change (in Figure 1B) was smaller in S_1_, which also increased the conjugated electronic structures. To conclude, drugs Alectinib and Rilpivirine exhibited the structural characteristics: planar parent skeleton, stronger π-conjugation effect in S_1_ and electron acceptors connecting conjugated structures, which would be a benefit for superior photo-physical performance.

### 2.2. Molecular Electrostatic Potential

**Figure 2:**
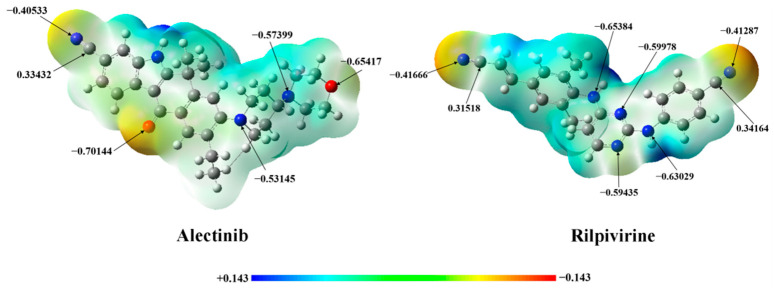
Molecular electrostatic potential surfaces plotted for the Alectinib and Rilpivirine (The blue and red regions represent electronic potential regions of positive and negative potential, respectively.).

The electrostatic potential is a representation of an electric charge distribution for a single molecule, which is an important property for binding with protein. The blue and red regions represent positive and negative electronic potential regions, respectively. The darker color is a “more positive” or “more negative” potential. A negative electric potential means that a positive charge group will be attracted easily. For Alectinib (shown in Figure 2), the red color indicates a higher electron density around the oxygen and cyan group representing that it will benefit the forming interaction with a positive charge group (such as amide group) in protein. On the other hand, the amino group of compounds Alectinib and Rilpivirine (shown in Figure 2) exhibit obvious positive electric potential, which will attract the negative charge group (such as carbonyl group) in protein.

### 2.3. UV-vis Experiments and One-Photon Absorption (OPA) Spectral Properties

To evaluate the UV-vis spectra of molecules Alectinib and Rilpivirine, we first tested the solvent-dependent excited spectra in organic solvent 100%DMSO (dimethyl sulfoxide), 50%DMSO-50%PBS (phosphate-buffered saline) and 10%DMSO-90%PBS (shown in Figure S1, Supplementary Materials). As displayed in Figure S1, the UV-vis spectra of Alectinib and Rilpivirine at the three solvent exhibited one maximum characteristic peak and were centered at about 345 nm and 310 nm, respectively. Upon adding PBS, the intensity of excited characteristic peak for Alectinib was decreased gradually, accompanied with a red-shift wavelength from 338 nm → 348 nm → 349 nm in Figure S1A. As well as for Rilpivirine, following with increased PBS, the intensity of absorption peak also was reduced from Figure S1B. It was worth noted that the solution became some turbid for Alectinib and Rilpivirine upon adding 25%PBS solvent in UV-vis experiments, which was in accordance with the reported poor/medium soluble for Alectinib and Rilpivirine [23]. The poor/medium soluble may lead to the reduced intensity of UV-vis spectra along with increased PBS proportion. To further explore the photo-physical properties of UV-vis spectra, theoretical calculation for OPA was performed in the next, which would be discussed in detail.

Spectra is closely related with optical properties and electronic characteristics. So, the simulated OPA spectral properties of Alectinib and Rilpivirine in water were obtained and listed in Table 2, concluding OPA wavelength ($\lambda_{max}^{OPA}$), oscillator strengths ($f^{\;O}$), vertical excitation energies ($E^{0f}$), transition dipole moment ($\mu^{0f}$) and transition characteristics. TDDFT methods were employed to obtain the OPA spectra according quantum chemical calculation. The solvation model density (SMD) [24,25] with default parameters of H_2_O was used to implicitly consider the homogeneous dielectric solvation effects. Additionally, compared with the results from Figure S1 and Table 2, we found that the calculated wavelengths of Alectinib and Rilpivirine were in reasonable agreement with the experimental results generally.

**Figure 3:**
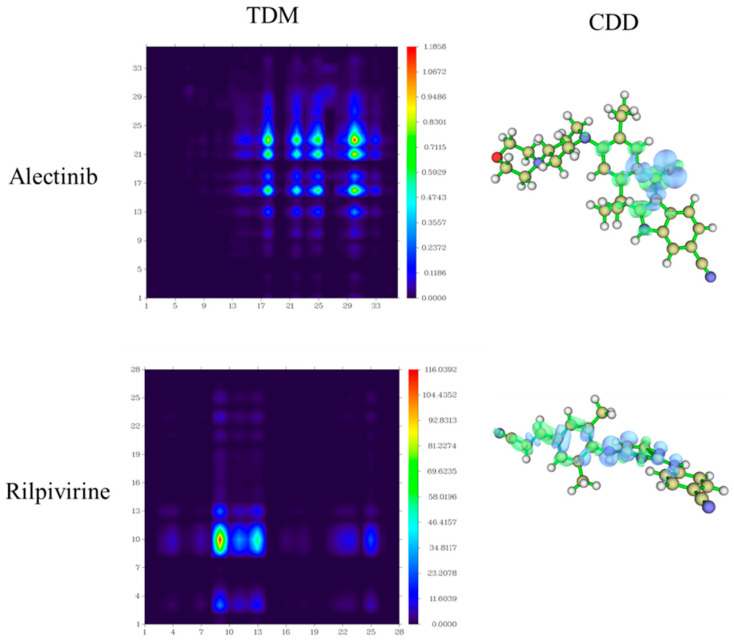
The transition density matrix (TDM) and charge density difference (CDD, green represented electron distribution and blue was hole distribution.) plots of Alectinib and Rilpivirine.

The main absorption peak (349^Exp.^ nm) in 10%DMSO-90%PBS of Alectinib was connected with the first excited state S_1_, which was derived from HOMO → LUMO transition (88.1%) and had localized excitation (LE) and charge transfer (CT) characteristics (see Figure S2). The CT characteristic mainly came from the 1-(2-ethylphenyl) piperidine group to the indole group due to the electron-accepting group (–CN) connecting with π-conjugated indole ring. For Rilpivirine, the maximum excited wavelength (311^Exp.^ nm) in 10%DMSO-90%PBS was also derived from S_0_ → S_1_ electron transition with a major contribution of HOMO-1 → LUMO (56.7%). Furthermore, the transition density matrix (TDM) and charge density difference (CDD) were adopted to describe the electron transfer process directly from the ground and excited state in the whole molecule [26,27,28]. Here, we mainly studied the electronic transition process of S_0_ → S_1_ for Alectinib and Rilpivirine in the OPA spectrum, and the electronic transition properties were analyzed qualitatively by TDM and CDD. In the meantime, more transition indexes were also listed in Table S2, including the centroid distance of the electrons and holes (D), electron-hole overlap index (Sr), average distribution breadth of the electrons and holes (H), hole delocalization index (HDI) and electron delocalization index (EDI) [29]. Firstly, the two-dimensional diagram showed the charge density difference, in which the green isosurface represented electron distribution while the blue isosurface represented hole distribution. In the transition process from the S_0_ to S_1,_ the molecule Alectinib showed the characteristic of local excitation and weak charge transfer, which was mainly concentrated on the 7-ethyl-4-methylnaphthalen-1(4*H*)-one group in Figure 3 (CDD). The smaller average distribution breadth (H: 2.038 Å) of the electrons and holes for Alectinib was due to the local excitation from 7-ethyl-4-methylnaphthalen-1(4*H*)-one group mainly. The centroid distance (D: 1.084 Å) of the electrons and holes for Alectinib was smaller, likely because of the weak charge transfer from the carbanyl group to 6-ethyl-1-methyl-1,4-dihydronaphthalene. For molecule Rilpivirine, owning to the existence of two cyano groups in the left terminal, larger local excitation occurred in the left half of the molecule and the electronic transfer happened from right to left. Combining with TDM plots in Figure 3, it could be seen that stronger contribution of electron transfer for Rilpivirine resulted from the 8th nitrogen atom in linker to the 10th carbon atom in benzene ring (atomic numbers in Figure S3) [30]. Lastly, the larger electron-hole overlap index (Sr: 0.733) in Rilpivirine implied the more electron-hole overlap, which was also seen in CDD plots.

### 2.4. TPA Spectral Properties

The traditional fluorescence imaging technique applyies one-photon microscopy (OPM), which involves the application of UV-vis light as an excitation source and achieves the lower tissue penetrations depth (generally 100 mm), limiting the application of OPM in living systems [31]. Over the past decades, the fluorescence imaging technique basing on multiphoton absorption uses near-infrared (NIR) as excitation sources, which has been proved to be one of the most effective tools in biomedical imaging applications [5,32]. For two-photon microscopy (TPM), the electron of fluorophore is excited to excited states after absorbing two photons simultaneously, using half the energy of photons compared to OPM [2]. Maria Goppert Mayer, as a Nobel laureate, first envisioned the concept of using two light quanta to excite a fluorophore. In her honor, the unit ‘GM’ was used to represent the TPA cross section values of a molecule [33]. Subsequently, the first cellular images was obtained by Webb et al. according to simultaneous excitation of fluorophore with two photons of NIR wavelength (700 nm–1100 nm) using femtosecond (*fs*) pulsed laser, which was in biological optical window to excite the fluorophore. The TPM technology has exclusive advantages in fluorescence imaging, including deeper imaging depth (down to 1 mm), less photobleaching, weaker background fluorescence, and higher spatiotemporal resolution [34]. These promising features have inspired more and more scientists to develop novel fluorescent molecules with enhanced TPA properties by designing molecules with an appropriate donor–acceptor systems, suitable dipolars, π-bridges, quadrupolars, octupolar characteristics and more [35,36,37]. Thus, the potential TPA properties of Alectinib and Rilpivirine were also predicted in this section, hoping to improve their bio-imaging applications.

As we all known, effective TPA imaging is influenced by the TPA cross-section ($\delta^{TPA}$) and fluorescent quantum yield (Φ) simultaneously. The $\delta^{TPA}$ denotes the TPA probability of a molecule. The larger the TPA cross-section, the larger the probability is for reaching the excited state after absorbing two photons simultaneously. In this work, we used the response function theory method to obtain the TPA properties [38,39]. We performed the calculation for the TPA spectral properties of both Alectinib and Rilpivirine, including the maximum TPA cross sections ($\delta_{max}^{TPA}$) and corresponding TPA wavelengths ($\lambda_{max}^{TPA}$) by DALTON software (Dalton2021.alpha, http://daltonprogram.org) in the 550 nm–1000 nm region [40]. Firstly, in order to decrease the deviation of simulated TPA spectra, two common TD-DFT functionals (Cam-B3LYP and B3LYP) for predicting the TPA properties were adopted here. The TPA spectra in gas and water (with PCM solvent) were obtained and listed in Table 3 and Table S3, using Cam-B3LYP and B3LYP functional, respectively. We could draw the following: (i) not only by B3LYP but also by Cam-B3LYP, the TPA wavelength and cross section of Alectinib in water was longer and larger than that in gas, such as 647.4 nm/159 GM (water) > 623.00 nm/1.0 GM (gas), suggesting the potential application in biological systems, as well as for compound Rilpivirine. (ii) Molecules Alectinib and Rilpivirine generally exhibited shorter TPA wavelength and smaller TPA cross section under Cam-B3LYP functional compared with those under B3LYP functional, which were in agreement with the reported investigation [12]. But the transition characteristic for the TPA spectra of Alectinib and Rilpivirine by the two functionals was consistent (from S_0_ → S_1_). Considering the reported better results from B3LYP compared with the experimental data, we adopted the calculated TPA properties by B3LYP functional for the later discussion [41,42]. (iii) In a water environment, the compound Alectinib exhibited a larger TPA cross-section ($\delta_{max}^{TPA}$: 269.0 GM) at 772.5 nm, which was in the NIR wavelength region. From the next fluorescence experiment, the fluorescence quantum yield of Alectinib was 7.5%, so its action TPA cross-section ($\delta_{max}^{TPA}$*Φ) was 201.75 GM, which was larger than 50 GM and was suitable for applications in biological samples with reasonable incident laser power [31]. (iv) For Rilpivirine, the TPA cross section in water was medium ($\delta_{max}^{TPA}$: 159.0 GM) at 744.6 nm excited wavelength, but the fluorescence quantum yield (Φ: 1.1%) was lower. Thus, its smaller action cross-section ($\delta_{max}^{TPA}$*Φ: 17.49 GM) might restrict the potential application in TPA bio-imaging. The latter sections were devoted to discussing important aspects that affected their TPA properties.

In order to further clarify the origin of the TPA activity of Alectinib and Rilpivirine and explain the calculated TPA spectra, the two-state approximation expression (X) related to the TPA cross section was adopted here [43,44]:

(1) $$\delta_{\max}^{\mathrm{TPA}}\propto \frac{{\left(M^{01}\right)}^{2}{\left(\left|\Delta \mu^{01}\right|\right)}^{2}}{{\left(E^{01}\right)}^{2}}$$

The values of transition/state dipole moment vectors and transition energy involved in two-state approximation model were listed in Table 4. As shown in Table 4, it was clear that the larger TPA cross-section of Alectinib resulted from the smaller transition energy ($E^{01}$ = 3.83 eV), larger transition state dipole moment (2.99 Debye), and bigger difference of state dipole moment ($\left|\Delta \mu^{01}\right|$ = 2.48 Debye) mainly. Additionally, the simulated TPA tensor elements basing on the quadratic response theory were also listed in Table 5 to reveal the structural characteristics for TPA properties of Alectinib and Rilpivirine. By using the TPA tensor elements in forma (4), the maximum TPA cross-sections in atomic units (a.u.) were obtained and displayed in Table 5. From Table 5, it could be seen that the Sxx component had a significant contribution in promoting the TPA process for Alectinib and Rilpivirine, which happened in the direction of ICT from Figure S2. Thus, the larger TPA cross-section for Alectinib might result from the bigger charge transfer amount (Alectinib (0.723 e) > Rilpivirine (0.546 e), in Table 3) during the TPA transition process.

### 2.5. Fluorescence Spectral Properties

**Figure 4:**
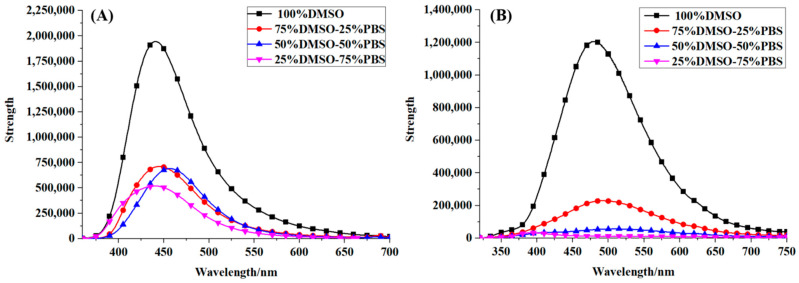
Fluorescence spectra of Alectinib (**A**) and Rilpivirine (**B**) with 100 μM by excitation at 340 nm and 310 nm, respectively.

For optical imaging and detection, it is essential to have high fluorescence efficiency. Basing on the systematical analysis of absorption spectral properties, how about the fluorescence properties of Alectinib and Rilpivirine? Thus, the fluorescence spectra of the molecules Alectinib and Rilpivirine in different solvents (100%DMSO, 75%DMSO-25%PBS, 50%DMSO-50%PBS and 25%DMSO-75%PBS) were measured by fluorescence experiments, respectively, and were drawn in Figure 4. As shown in Figure 4, we noticed that the emission wavelength of Alectinib and Rilpivirine were about 450 nm and 500 nm, respectively, and the fluorescence efficiency of Alectinib was higher than that of Rilpivirine (Φ: 7.5% > 1.1%). Furthermore, the fluorescence intensity in 100%DMSO was strongest for the two compounds, and it exhibited an obvious decreasing tendency along with the increase in PBS content. What is the reason? Of particular note was that in the fluorescent experiments, the solution became more turbid for the compounds Alectinib and Rilpivirine upon adding 25%PBS, which was in agreement with the reported poor/medium soluble [23]. Thus, the fluorescence spectra were further obtained in different concentrations for Alectinib and Rilpivirine, respectively, which were displayed in Figure S4. It could be clearly seen that, following the increased sample concentration (50 μM → 100 μM → 150 μM), the emission intensity was increased gradually. Those demonstrated that the decreased fluorescent intensities of Alectinib and Rilpivirine in more PBS proportion were due to their lower solubility. Additionally, there was a red shift of fluorescence peak following with the increased sample concentration in Figure S4. Most of the chemo drugs benefited the florescence properties due to their aromatic rings. The red shift of fluorescence peak took place at a dense solution due to the photon reabsorption effects when the stokes shift between absorption/fluorescence spectra was sufficiently small as it happened for the chemo drugs of interest here [45,46]. According to Figure S4, the small Stokes shift lucidly appeared that was the origin of photon reabsorption effects and subsequent red shift. To explore the origin of these emission spectra, the theoretical study was analyzed next.

To deeply explore the origins of the fluorescence properties about these two molecules, we adapted the following TD-DFT//B3LYP/6-31+G(d) calculations and obtained the detailed excited properties. As we all known, the fluorescence quantum yield (Φ) is an important index for measuring fluorescence efficiency, which is determined by the radiative decay rate ($K_{r}$) and the nonradiative decay rate ($K_{nr}$) theoretically. According to Kasha’s rule, the electron can transfer from the first singlet excited state (S_1_) to the ground state (S_0_) through the radiative and non-radiative decay processes. Thus, we thoroughly explored the S_1_ excited-state properties for the compounds Alectinib and Rilpivirine, and the results including emission wavelength (λ^EMI^), oscillator strengths (*f^E^*), transition dipole moment ($\mu_{EMI}^{f0}$), charge transfer quantity ($q_{EMI}^{CT}$), charge transfer distance ($d_{EMI}^{CT}$) and transition characteristics, and were listed in Table 6. It could be found that the maximum emission wavelength of Alectinib was at 456.7 nm (450 nm ^Exp.^), which was originated from the S_1_ → S_0_ electron transition with a major contribution of LUMO→HOMO (98.3%). For Rilpivirine, its maximum emission wavelength was also derived from the electron transition S_1_ → S_0_, which was mainly constructed by LUMO→HOMO (97.6%).

**Figure 5:**
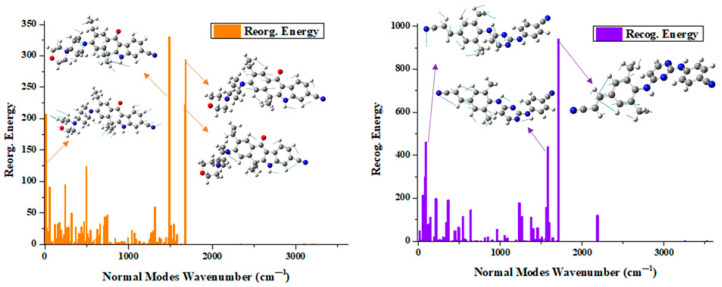
Reorganization energies (cm^−1^) and crucial displacement vectors for the normal modes with large reorganization energies for compounds Alectinib (origin) and Rilpivirine (purple).

From above fluorescence experiment, Alectinib had a larger fluorescence quantum yield than Rilpivirine (Φ: 7.5% > 1.1%) which might come from the larger electronic transition dipole moment and charge transition quantity during the emission process in Table 6 ($\mu_{EMI}^{f0}$: 8.41 Debye > 6.33 Debye, $q_{EMI}^{CT}$: 0.682 e > 0.502 e). Additionally, we also analyzed this phenomenon by means of reorganization energy, which was an important parameter for evaluating geometric relaxation and energy component in internal conversion process. From Figure 5, it could be summarized that (i) the total reorganization energy of Alectinib was smaller than that of Rilpivirine (Reorg. Energy: 2821.6 cm^−1^ < 5176.9 cm^−1^), implying the smaller geometric relaxation for Alectinib. (ii) In Alectinib, the four main vibration models (> 200 cm^−1^), including molecular skeleton scissoring vibration mode at 13.62 cm^−1^, benzene ring stretching vibration mode at 1486.36 cm^−1^, 1676.46 cm^−1^ and 1681.77 cm^−1^, made the contributions to the total reorganization energy. The C-O bond of benzene ring stretching vibration mode at 1486.36 cm^−1^ had a higher reorganization energy up to 330.34 cm^−1^, and occupied 11.71% of the total reorganization energy. (iii) For the molecule Rilpivirine, in the high-frequency regions, the C-C bond of the benzene ring attached to a double bond stretching vibration mode at 1708.87 cm^−1^ exhibited particular large reorganization energy up to 940.95 cm^−1^, which contributed 55.06% to its total reorganization energy. Concluding, compared to the compound Rilpivirine, molecule Alectinib possessed larger electronic transition dipole moment and charge transition quantity, which were beneficial for the radiative decay process, and the smaller geometric relaxation, which weakened the nonradiative decay process. On the other hand, the molecular aromatic rings had important influences on the fluorescence properties [39,41,47]. The Alectinib possessed stronger molecular planarity due to the appropriate aromatic ring substitution than that of Rilpivirine (46.6° < 111.0°), which also contributed the higher fluorescence quantum yield. Thus, Alectinib had higher fluorescence quantum yield than Rilpivirine in organic solvent. Lastly, how were their fluorescence properties in cell imaging?

### 2.6. Cell Imaging Application

**Figure 6:**
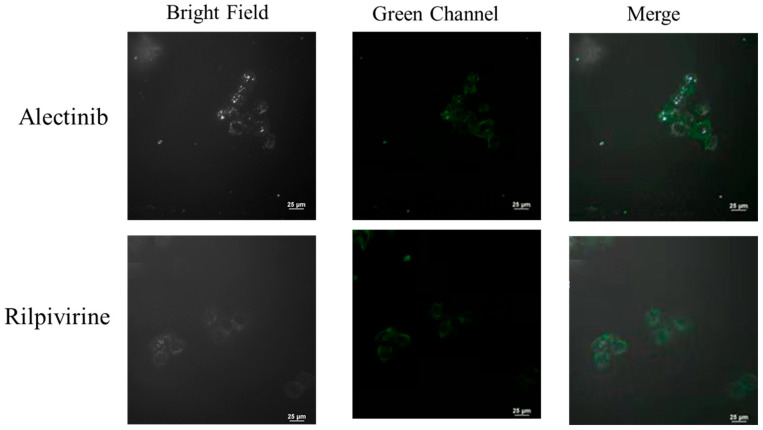
Confocal microscopy colocalization images of the studied drug molecules Alectinib and Rilpivirine in HeLa cells.

To evaluate the bio-imaging ability, the compounds Alectinib and Rilpivirine to stain living cells of HeLa cell line were studied by confocal laser scanning fluorescence microscopy. HeLa cells were cultured onto glass-bottom Petri dishes for 12 h before treatment. Live cells were treated with the compounds Alectinib and Rilpivirine at 20 μM for 16 h, washed with PBS three times, fixed in 4% paraformaldehyde solution for 15 min, and washed with PBS again, respectively. Cell images were obtained by confocal laser scanning fluorescence microscopy (CLSM, Nikon, Ti2-E+A1, Japan) using both 405 nm and 488 nm as the excitation wavelengths. As displayed in Figure 6, HeLa cells stained with the compounds Alectinib and Rilpivirine showed green fluorescence. It suggested that Alectinib and Rilpivirine had better imaging performances in cell imaging, although Rilpivirine had a lower fluorescence quantum yield than Alectinib (1.1% < 7.5%) in an organic solvent. These might come from the complex cellar environments, which formed some interaction with small molecules and enhanced the fluorescence intensity of Rilpivirine. So, Alectinib and Rilpivirine had the potential abilities for biological imaging applications. Compared with commercial dyes or drugs sharing with a single function, drug molecules Alectinib and Rilpivirine have potential practical applications in integrated diagnosis and treatment.

## 3. Material and Methods

In this work, the optimization and frequency calculations of ground-state and geometries structures for Alectinib and Rilpivirine were obtained at the level of M06-2X/6-31+G(d) with the help of Gaussian 16 program package [48,49]. Furthermore, their stable molecular geometries were displayed in Table 1. The three smallest vibrational frequencies of Alectinib and Rilpivirine at the real local minima points were positive values, confirming the stabilities of studied Alectinib and Rilpivirine geometries. The one-photon absorption (OPA) and emission spectral properties were simulated according to the time-dependent density functional theory (TD-DFT) based on stable molecular structures. The M06-2X/6-31+G(d) and B3LYP/6-31+G(d) level were used for OPA and emission spectral simulation, respectively. At the same time, the solvent effect was taken into account within the self-consistent reaction field (SCRF) theory through applying SMD model [24]. On the other hand, the two-photon absorption (TPA) properties were calculated with the help of quadratic response theory in the DALTON program [40]. The reorganization energies with the parameters of normal mode displacements were carried out by the MOMAP program [50,51].

The OPA transition probability ($\delta^{OPA}$) is able to evaluate the OPA intensity. The OPA oscillator strength (*f*), as an important index for measuring various OPA property of a fluorescent compound, is related to $\delta^{OPA}$. The expression of $\delta^{OPA}$ from the ground state S_0_ to the excited state Sn is obtained by [52]:

(2) $$\delta^{OPA}=\frac{2\omega_{n}}{3}\sum_{\alpha }{\left|\langle 0|\hat{\mu_{\alpha }}|n\rangle \right|}^{2}\;\left(\alpha \;\in x,\;y,\;z\right)$$

here, $\omega_{n}$ represents the excited energy. $\langle 0|\hat{\mu_{\alpha }}|n\rangle$ is the transition dipole moment along with the different coordinate directions, which is related to the wave function integral. These physical parameters about the OPA spectra can be calculated with the help of Gaussian 16 program [49].

The TPA cross-section ($\delta^{TPA}$) is used to evaluate TPA intensity, which is determined by two-photon transition probability ($\sigma^{TPA}$) and can be given by [53]:

(3) $$\delta^{TPA}=\frac{4\pi^{2}\alpha \alpha_{0}^{5}\omega^{2}}{15c\Gamma }\sigma^{TPA}$$

here, α, $\alpha_{0}$ and c are on behalf of the fine structure constant, Bohr radius, and the speed of light, respectively; ω represents the photon energy in atomic units, and Γ is the broadening factor, describing spectral broadening of an excitation, which has been assumed to be 0.05 eV to make the theoretical simulation process consistent with the experimental spectra. $\sigma^{TPA}$ can be calculated as [54]:

(4) $$\sigma^{TPA}=\frac{1}{30}\sum_{ab}\left(FS_{aa}{\bar{S}}_{bb}+GS_{ab}{\bar{S}}_{ab}+HS_{ab}{\bar{S}}_{ba}\right)$$

*a*, *b* $\in \;\left\{x,\;y,\;z\right\}$, and F, G and H are 2, 2, and 2 for linearly polarized light and were −2, 3 and 2 for the circular case, respectively. Taking electric dipole approximation into consideration, the TPA transition tensor $S^{if}$ between the initial state *i* and the final state *f* can be expressed as [53]:

(5) $$S_{ab}^{if}=\underset{n\neq i}{\sum }\left\{\frac{\langle i|\mu_{\alpha }|n\rangle \langle n|{\bar{\mu }}_{b}|f\rangle }{\omega_{in}-\omega_{1}}+\frac{\langle i|\mu_{b}|n\rangle \langle n|{\bar{\mu }}_{a}|f\rangle }{\omega_{in}-\omega_{2}}\right\}$$

where $\langle i|\mu_{\alpha }|n\rangle$ is the *a*th compound of the transition dipole moment between the initial electric state *i* and the intermediate state *n*. $\omega_{in}$ is excitation energy. In addition, $\omega_{1}$ and $\omega_{2}$ presents the energies of two photons. These physical parameters about the TPA spectra can be obtained by quadratic response theory with the help of DALTON software [40].

Fluorescence intensity is determined by the competition of radiative decay and non-radiative decay process [42,55]. The fluorescence efficiency could be evaluated by fluorescence quantum yield (Φ), which was obtained from experiment directly in this investigation. The stronger of radiative decay, the larger of the Φ. Conversely, the smaller the non-radiative decay process, the larger of the Φ. Internal conversion (IC), as a non-radiative decay from the first excited state to ground state, is the most important component during the non-radiative process [56]. The IC rate can be calculated under harmonic oscillator approximation though Fermi’s golden rule and is expressed as [57]:

(6) $$K_{ic}=\frac{2\pi }{\hbar }{\left|H_{fi}^{\prime }\right|}^{2}\delta \left(E_{fi}+E_{f\nu_{f}}-E_{f\nu_{i}}\right)$$

The $K_{ic}$ is closely related to electron–vibration coupling and geometric relaxation of the excited state, which can be evaluated by reorganization energy (λ) or Huang−Rhys factor (HR). These above parameters of fluorescence spectra can be calculated by applying Gaussian 16 and MOMAP program [49,51].

The UV-vis excitation and fluorescence spectroscopy were carried out at room temperature and by the instrument of SHIMADZU 2550 UV–vis spectrophotometer (SHIMADZU, Kyoto, Japan) and SHIMADZU RF-6000 Fluorolog instrument (SHIMADZU, Kyoto, Japan), respectively. The slit bandwidth and sampling interval for Shimadzu RF-600 was 5 nm and 0.5 nm, respectively. Cell images were obtained by confocal laser scanning fluorescence microscopy (CLSM, Nikon, Ti2-E+A1, Tokyo, Japan) using 405 nm and 488 nm as the excitation wavelength. The HeLa cells were human cervical cancer cells in DMEM and EMEM culture medium from Human Fenghui Biotechnology Co. Ltd. (Nanjing, China), and its accession number was CL0134. HeLa cells were cultured onto glass-bottom Petri dishes for 12 h before treatment. Live cells were treated with Alectinib and Rilpivirine at 20 μM for 16 h, washed with PBS for 3 times, fixed in 4% paraformaldehyde solution for 15 min, and washed with PBS again.

## 4. Conclusions

In conclusion, the optical properties of two drugs Alectinib and Rilpivirine were deeply explored firstly, through the simulation of molecular structures, electrostatic potential, OPA/TPA and emission spectral properties, and experiment of UV-vis spectra, fluorescence and cell imaging. Moreover, the relationships between molecular structures and optical properties for Alectinib and Rilpivirine have been minutely investigated based on molecular modeling. The results suggested that the drugs Alectinib and Rilpivirine with planar parent skeleton, stronger π-conjugation effect in S_1_ and electron acceptors connecting conjugated structures could be excited by UV-vis spectra, and subsequently emitted fluorescence at 450 nm and 500 nm, respectively. It was noted that the intensities of UV-vis excited and emission spectra for Alectinib and Rilpivirine were decreased during the increased PBS proportion, which were ascribed to the poor/medium solubility. Meanwhile, we found that the fluorescence quantum yield of Alectinib was higher than that of Rilpivirine (Φ: 7.5% > 1.1%) in organic solvent, which might come from the larger electronic transition dipole moment ($\mu_{EMI}^{f0}$: 8.41 Debye > 6.33 Debye) and charge transition quantity ($q_{EMI}^{CT}$: 0.682 e > 0.502 e) during the emission process. Additionally, the smaller geometric relaxation of Alectinib due to the lower reorganization energy than Rilpivirine (2821.6 cm^−1^ < 5176.9 cm^−1^) weakened the non-radiative decay process, and also led to its higher fluorescence intensity. For TPA properties, we found that the action TPA cross-section ($\delta_{max}^{TPA}$*Φ) of Alectinib was 201.75 GM at 772.5 nm excited wavelength, which was larger than 50 GM in NIR region and was suitable for applications in biological samples with reasonable incident laser power [31]. Lastly, Alectinib and Rilpivirine displayed the same green fluorescence in HeLa cells, suggesting their potential bio-imaging applications. We hope that this investigation can provide useful guidance for the design and synthesis of more excellent fluorescent activated molecules.

## Figures and Tables

**Table 1:** The chemical structure, stable geometries of the ground state (S_0_) and first excited state (S_1_) for compounds Alectinib and Rilpivirine.

Molecules	S_0_	S_1_
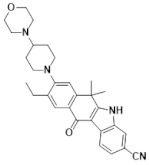 Alectinib	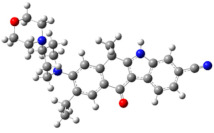 Top view 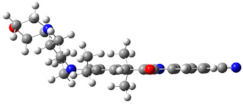 Side view	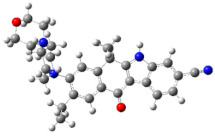 Top view 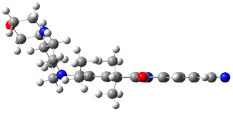 Side view
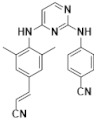 Rilpivirine	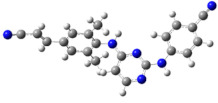 Top view 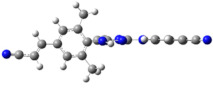 Side view	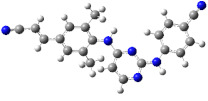 Top view 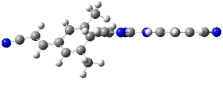 Side view

**Table 2:** Calculated OPA spectra properties of Alectinib and Rilpivirine in water concluding OPA wavelength (λ^OPA^), oscillator strengths ($f^{\;O}$), vertical excitation energies ($E^{0f}$), transition dipole moment ($\mu^{0f}$) and transition characteristics.

Molecule	$\lambda_{max}^{OPA}$/nm	$f^{\;O}\;$	$E^{0f}/\mathrm{eV}$	$\mu^{0f}/\mathrm{Debye}$	Transition Characteristics
Alectinib	318.0 ^a^ (349 ^Exp.^)	0.7456 ^a^	3.90 ^a^	1.1 ^a^	S_0_ → S_1_ (HOMO → LUMO 88.1%) ^a^
Rilpivirine	298.6 ^a^ (311 ^Exp.^)	1.4836 ^a^	4.15 ^a^	1.5 ^a^	S_0_ → S_1_ (HOMO-1 → LUMO 56.7%) ^a^

^a^—was the data from the calculated OPA properties. ^Exp.^—represented the data from experiments.

**Table 3:** The calculated TPA properties of Alectinib and Rilpivirine including the maximum TPA cross-section ($\delta_{max}^{TPA}$), corresponding TPA wavelength ($\lambda_{max}^{TPA}$), transition nature and charge transfer amount in gas (a) and water (b) by B3LYP functional.

Molecules	$\delta_{max}^{TPA}/\mathrm{GM}$	$\lambda_{max}^{TPA}/\mathrm{nm}$	Transition Nature	$q_{TPA}^{CT}/e$
Alectinib	44.8 ^a^ 269.0 ^b^	700.5 ^a^ 772.5 ^b^	S_0_→S_1_^a^ (HOMO → LUMO) S_0_→S_1_^b^ (HOMO → LUMO)	0.723 ^b^
Rilpivirine	21.8 ^a^ 159.0 ^b^	756.0 ^a^ 744.6 ^b^	S_0_→S_1_^a^ (HOMO → LUMO) S_0_→S_1_^b^ (HOMO → LUMO)	0.546 ^b^

^a^ were the calculated TPA properties in gas. ^b^ represented the calculated TPA properties in water.

**Table 4:** Parameters related to TPA transition process of Alectinib and Rilpivirine.

Molecules	Excited State	$\mu^{00}/\mathrm{Debye}$	$\mu^{11}/\mathrm{Debye}$	$\left|\Delta \mu^{01}\right|/\mathrm{Debye}$	$M^{01}/\mathrm{Debye}$	$E^{01}/\mathrm{eV}$
Alectinib	S_1_	10.96	13.44	2.48	2.99	3.83
Rilpivirine	S_1_	9.32	10.54	1.22	2.00	3.99

**Table 5:** TPA tensor elements ($S_{ab}$) and TPA cross sections ($\sigma^{TPA}$) (in au) for Alectinib and Rilpivirine molecules calculated in water solvent by DALTON software with B3LYP functional.

Molecules	Excited State	$S_{ab}/a.u.$						$\sigma^{TPA}/a.u.$
		$S_{xx}$	$S_{yy}$	$S_{zz}$	$S_{xy}$	$S_{xz}$	$S_{yz}$	
Alectinib	S_1_	422.7	−31.7	7.5	6.6	63.2	−1.0	1,068,860.6
Rilpivirine	S_1_	286.6	18.5	−2.9	2.5	4.7	95.6	590,613.0

**Table 6:** Fluorescence quantum yield (Φ) from experiments and calculated emission spectra properties of Alectinib and Rilpivirine concluding emission wavelength (λ^EMI^), oscillator strengths (*f^E^*), transition dipole moment ($\mu_{EMI}^{f0}$), charge transfer quantity ($q_{EMI}^{CT}$), charge transfer distance ($d_{EMI}^{CT}$) and transition characteristics using B3LYP functional and 6-31+G(d) basis set.

Molecule	λ^EMI^/nm	*f* *^E^*	$\mu_{EMI}^{f0}/\mathrm{Debye}$	$q_{EMI}^{CT}/e$	$d_{EMI}^{CT}/Å$	Transition Characteristics	Φ/%^Exp^
Alectinib	456.7 450^Exp.^	0.7469	8.41	0.682	2.568	S_1_ → S_0_ (LUMO → HOMO 98.3%)	7.5
Rilpivirine	435.4 500^Exp.^	1.3784	6.33	0.502	2.627	S_1_ → S_0_ (LUMO → HOMO 97.6%)	1.1

^Exp.^ referred to the results from fluorescence experiments.

## Data Availability

Data available on request from the authors.

## Supplementary Materials

The following supporting information can be downloaded at: https://www.mdpi.com/article/10.3390/molecules28166172/s1. Tables S1–S4: The main selected bond length (Å) and dihedral angles (°) at the optimized S_0_ and S_1_ geometries for compounds Alectinib and Rilpivirine. Transition index of Alectinib and Rilpivirine from S_0_ to S_1_ in OPA spectra, including centroid distance of the electrons and holes (D), electron-hole overlap index (Sr), average distribution breadth of the electrons and holes (H), hole delocalization index (HDI) and electron delocalization index (EDI). Calculated TPA properties including the maximum TPA cross-section ($\delta_{max}^{TPA}$), corresponding TPA wavelength ($\lambda_{max}^{TPA}$), and transition nature of Alectinib and Rilpivirine in gas (a) and water (b) by Cam-B3LYP functional. The transition energy gaps (ETEG) of Alectinib in H2O and DMSO (polarity: H_2_O > DMSO).; Figure S1–S5: The UV-vis spectra of molecule Alectinib (A) and Rilpivirine (B) with the concentration of 100 μM in different solvent (100%DMSO, 50%DMSO-50%PBS and 10%DMSO-90%PBS). Contour surfaces of eight frontier molecular orbitals of Alectinib and Rilpivirine. Atomic numbers for compounds Alectinib and Rilpivirine. Fluorescence spectra of Alectinib (A) and Rilpivirine (B) in 25%DMSO-75%PBS solvent by excitation at 340 nm and 310 nm, respectively. UV-vis spectra of Alectinib (C) and Rilpivirine (D) in 25%DMSO-75%PBS solvent. The plot of fluorescence intensity (red) and wavelength (black) versus concentration for Alectinib (A) and Rilpivirine (B) in 25%DMSO-75%PBS solvent [58].

## Sample Availability

Samples of the compounds Alectinib and Rilpivirine are available from the authors.
