# Supplementary material for: Photophysical Exploration of Alectinib and Rilpivirine: Insights from Theory and Experiment
Source: Molecules. 2023 Aug 21;28(16):6172. doi: 10.3390/molecules28166172 (PMC10458258; doi:10.3390/molecules28166172)
Supplement: Supplementary file 1 [file molecules-28-06172-s001.zip › molecules-2501637-SI.pdf]

Supporting Information

Photophysical Exploration of Alectinib and Rilpivirine: Insights from Theory and Experiment

Chun Zhang [1], Yuting Yang [1], Suya Gan [1], Aimin Ren [2], Yu-Bo Zhou [3,4], Jia Li [3,4], Da-Jun Xiang [5,*] and Wen-Long Wang [1,*]

[1] School of Life Sciences and Health Engineering, Jiangnan University, Wuxi 214122, China
[2] Institute of Theoretical Chemistry, College of Chemistry, Jilin University, Liutiao Road 2#, Changchun 130061, China
[3] National Center for Drug Screening, State key Laboratory of Drug Research, Shanghai Institute of Materia Medica, Chinese Academy of Sciences, Shanghai 201203, China
[4] Zhongshan Institute for Drug Discovery, Shanghai Institute of Materia Medica, Chinese Academy of Sciences, SSIP Healthcare and Medicine Demonstration Zone, Zhongshan Tsuihang New District, Zhongshan 528400, China
[5] Xishan People's Hospital of Wuxi City, Wuxi 214105, China
[*] Correspondence: xiangdjxshospital@yeah.net (D.-J.X.); wenlongwang@jiangnan.edu.cn (W.-L.W.)

Table of Contents

Table S1. The main selected bond length (Å) and dihedral angles (°) at the optimized S_0 and S_1 geometries for compounds Alectinib and Rilpivirine.
Figure S1. The UV-vis spectra of molecule Alectinib (A) and Rilpivirine (B) with the concentration of 100 μM in different solvent (100%DMSO, 50%DMSO-50%PBS and 10%DMSO-90%PBS).
Figure S2. Contour surfaces of eight frontier molecular orbitals of Alectinib and Rilpivirine.
Table S2. Transition index of Alectinib and Rilpivirine from S_0 to S_1 in OPA spectra, including centroid distance of the electrons and holes (D), electron-hole overlap index (Sr), average distribution breadth of the electrons and holes (H), hole delocalization index (HDI) and electron delocalization index (EDI).
Figure S3. Atomic numbers for compounds Alectinib and Rilpivirine.
Table S3. Calculated TPA properties including the maximum TPA cross-section (δ_{max}^{TPA}), corresponding TPA wavelength (λ_{max}^{TPA}), and transition nature of Alectinib and Rilpivirine in gas (a) and water (b) by Cam-B3LYP functional.
Figure S4. Fluorescence spectra of Alectinib (A) and Rilpivirine (B) in 25%DMSO-75%PBS solvent by excitation at 340 nm and 310 nm, respectively. UV-vis spectra of Alectinib (C) and Rilpivirine (D) in 25%DMSO-75%PBS solvent.
Figure S5. The plot of fluorescence intensity (red) and wavelength (black) versus concentration for Alectinib (A) and Rilpivirine (B) in 25%DMSO-75%PBS solvent.
Table S4. The transition energy gaps (E_{TEG}) of Alectinib in H_2O and DMSO (polarity: H_2O > DMSO).

Table S1. The main selected bond lengths (Å) and dihedral angles (°) at the optimized S_0 and S_1 geometries for compounds Alectinib and Rilpivirine.

Bond/Dihedral Angle	Alectinib		Bond/Dihedral Angle	Rilpivirine	
	S_0	S_1		S_0	S_1
N29-C30 (Å)	1.40772 [a]<br>1.40672 [b]	1.36606 [a]	C33-C30 (Å)	1.42788 [a]<br>1.41919 [b]	1.39869 [a]
C36-C48 (Å)	1.48246 [a]<br>1.48020 [b]	1.43901 [a]	C30-C29 (Å)	1.34587 [a]<br>1.35301 [b]	1.41099 [a]
C48-O49 (Å)	1.24186 [a]<br>1.24940 [b]	1.29586 [a]	C29-C18 (Å)	1.46501 [a]<br>1.45898 [b]	1.40106 [a]
C46-C48 (Å)	1.44293 [a]<br>1.44504 [b]	1.43666 [a]	C13-N9 (Å)	1.41889 [a]<br>1.42111 [b]	1.36365 [a]
C45-C46 (Å)	1.38596 [a]<br>1.38991 [b]	1.39957 [a]	N9-C3 (Å)	1.36459 [a]<br>1.36926 [b]	1.38352 [a]
C32-C30-N29-C22 (°)	61.2 [a]<br>59.6 [b]	46.6 [a]	C14-C13-N9-C3 (°)	145.8 [a]<br>144.5 [b]	111.0 [a]

a represented the calculated data from M06-2X/6-31+G(d), b was the calculated results from B3LYP/6-31G(d,p).

The main selected bond lengths (Å) and dihedral angles (°) at the optimized S_0 of two drugs with the help of B3LYP/6-31G(d,p) were displayed in Table S1. It could be seen that the differences from M06-2X/6-31+G(d) and B3LYP/6-31G(d,p) method were minimal, for example the bond length (N29-C30) in Alectinib was 1.40772 Å and 1.40672 Å, respectively, implying that the optimized structures from M06-2X/6-31+G(d) were reasonable.

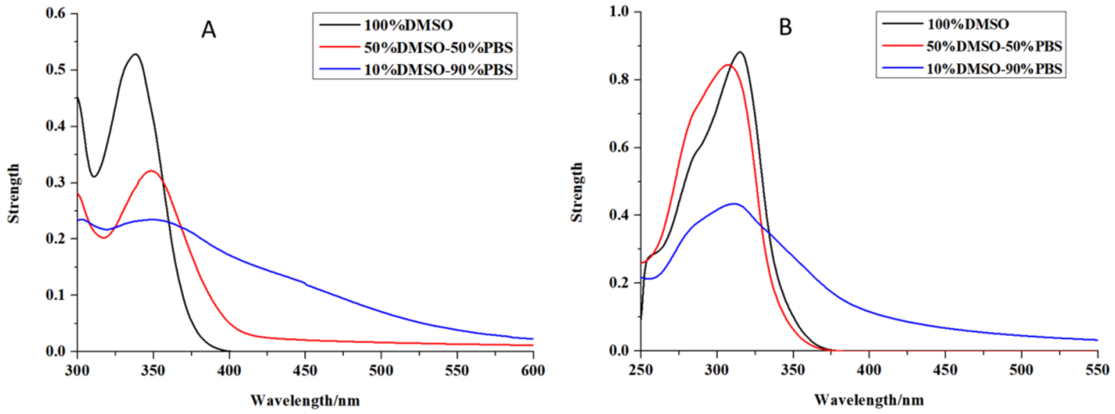

Figure S1. The UV-vis spectra of molecule Alectinib (A) and Rilpivirine (B) with the concentration of 100 μM in different solvent (100%DMSO, 50%DMSO-50%PBS and 10%DMSO-90%PBS).

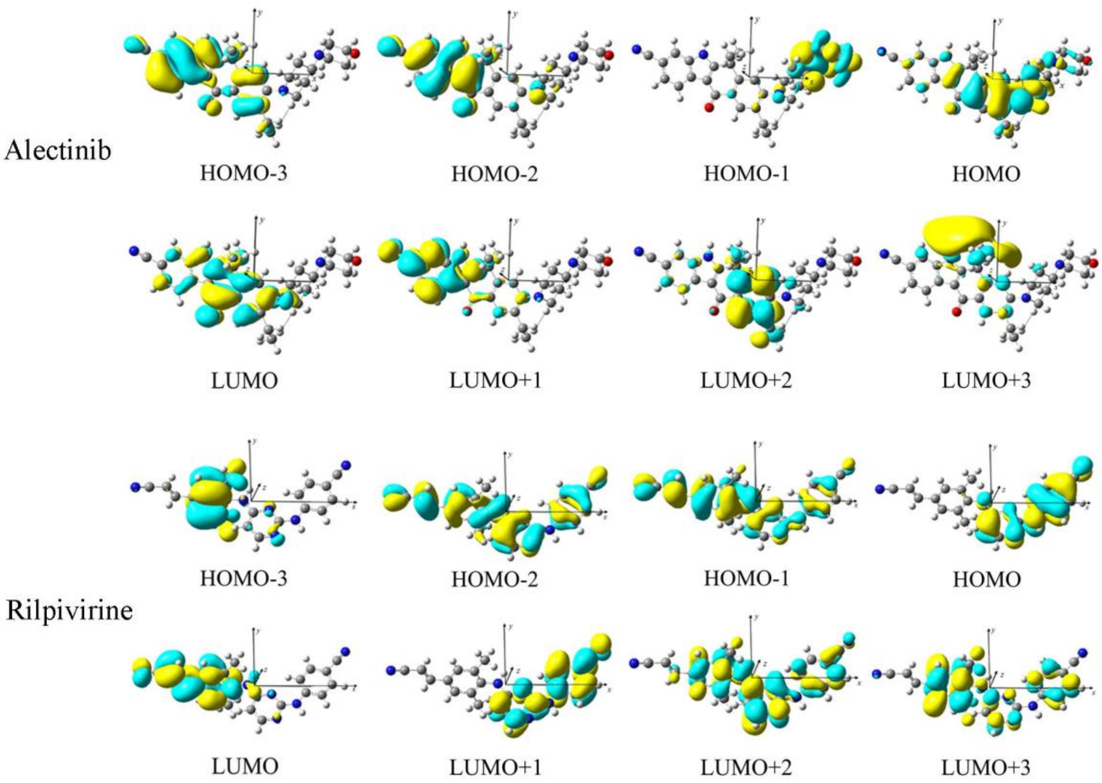

Figure S2. Contour surfaces of eight frontier molecular orbitals of Alectinib and Rilpivirine.

Table S2. Transition index of Alectinib and Rilpivirine from S_0 to S_1 in OPA spectra, including centroid distance of the electrons and holes (D), electron-hole overlap index (Sr), average distribution breadth of the electrons and holes (H), excitation energy from ground state to excited state (E), hole delocalization index (HDI) and electron delocalization index (EDI).

**Molecules**		**D (Å)**	**Sr**	**H(Å)**	**t(Å)**	**HDI**	**EDI**
Alectinib	S_1	1.084	0.503	2.038	-0.156	23.99	12.06
Rilpivirine	S_1	1.859	0.733	4.221	-1.856	6.64	5.91

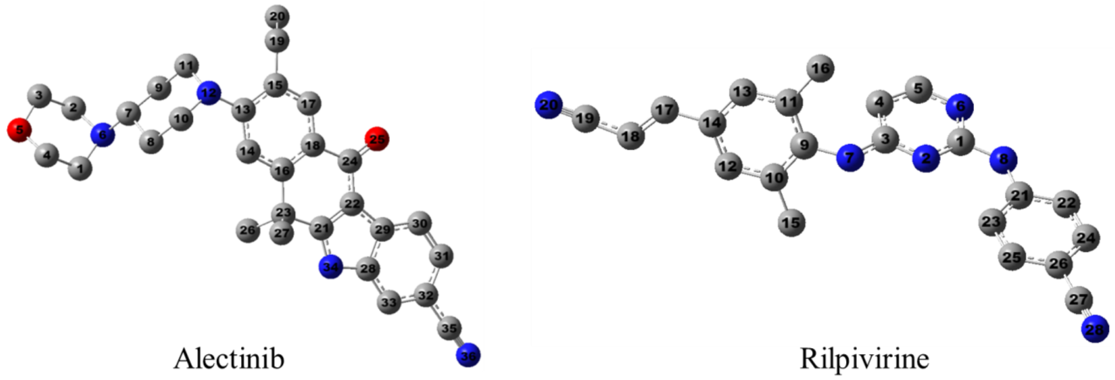

Figure S3. Atomic numbers for compounds Alectinib and Rilpivirine.

Table S3. Calculated TPA properties including the maximum TPA cross-section (δ_{max}^{TPA}), corresponding TPA wavelength (λ_{max}^{TPA}), and transition nature of Alectinib and Rilpivirine in gas (a) and water (b) by Cam-B3LYP functional.

Molecules	δ_{max}^{TPA}/GM	λ_{max}^{TPA}/nm	Transition nature
Alectinib	1.0^a	623.0^a	$S_0 \rightarrow S_1^a$ (HOMO → LUMO)
	159.0^b	647.4^b	$S_0 \rightarrow S_1^b$ (HOMO → LUMO)
Rilpivirine	29.3^a	582.1^a	$S_0 \rightarrow S_1^a$ (HOMO → LUMO)
	98.0^b	621.5^b	$S_0 \rightarrow S_1^b$ (HOMO → LUMO)

a were the calculated TPA properties in gas. b represented the calculated TPA properties in water.

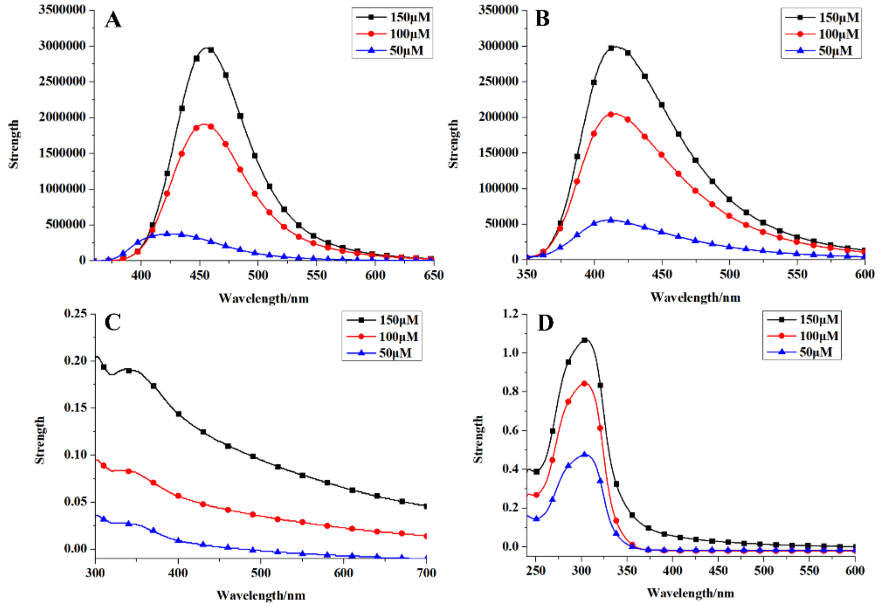

Figure S4. Fluorescence spectra of Alectinib (A) and Rilpivirine (B) in 25%DMSO-75%PBS solvent by excitation at 340 nm and 310 nm, respectively. UV-vis spectra of Alectinib (C) and Rilpivirine (D) in 25%DMSO-75%PBS solvent.

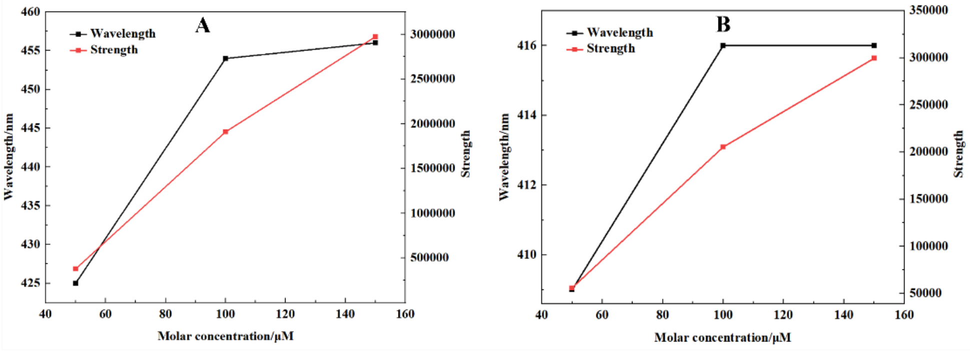

Figure S5. The plot of fluorescence intensity (red) and wavelength (black) versus concentration for Alectinib (A) and Rilpivirine (B) in 25%DMSO-75%PBS solvent.

In Figure S4 (A, B) and S5, along with the increased concentration of Alectinib and Rilpivirine, the effective amount molecules in solvent were increased which contributed to the elevated fluorescence intensity. The longer emission wavelength

was likely caused by the molecular aggregation effect following the increased concentration. (*Molecules,* **2020**, *25(3)*, 585.) As well as the fluorescence intensity, the UV-vis spectra intensity (in Figure S4 (C, D)) was increased along with the increased concentration (50 μM → 100 μM → 150 μM). On the other hand, the decreased fluorescence intensity of Alectinib (in Figure 4) was due to its poor water-solubility mainly during the increased proportion of PBS water solvent. It was agreement with the results from Figure S4 (the weaker fluorescence along with the lower concentration). We further got the transition energy gaps for Alectinib in H_2O and DMSO solvent (polarity: H_2O > DMSO) (in Table S4). It demonstrated that the blue-shift (455 nm → 445 nm) from 100%DMSO to 25%DMSO-75%PBS solvent in Figure 4 might be resulted from the larger transition energy gap (E_{TEG}: 6.05 eV (H_2O) > 5.95 eV (DMSO) in stronger polarity solvent.

Table S4. The transition energy gaps (E_{TEG}) of Alectinib in H_2O and DMSO (polarity: H_2O > DMSO).

Molecules	E_{TEG} /eV in H_2O	E_{TEG} /eV in DMSO	ΔE/eV
Alectinib	6.05	5.95	0.10
